# Editorial: Online data collection for human neuroscience: challenges and opportunities

**DOI:** 10.3389/fnhum.2024.1440711

**Published:** 2024-06-25

**Authors:** Colleen Mills-Finnerty, Peter J. Bayley, James O. Clifford, J. Wesson Ashford

**Affiliations:** ^1^VA Palo Alto Health Care System, Veterans Health Administration, United States Department of Veterans Affairs, Palo Alto, CA, United States; ^2^Division of Public Mental Health and Population Sciences, Department of Psychiatry and Behavioral Sciences, School of Medicine, Stanford University, Stanford, CA, United States; ^3^Department of Psychology, College of San Mateo, San Mateo, CA, United States

**Keywords:** cognitive assessment, online data collection, methods, neuroscience, aging

The COVID-19 pandemic forced an unprecedented shift toward remote data collection, and this increased adoption rate accelerated the pace of methodological development. Through the use of browser and phone app-based data collection methods, home devices for measuring behavioral activity and physiological data, and wider adoption of telehealth, participating in research has been made more accessible through technology. The rapid expansion in the use of such technologies makes it possible to digitally transmit participant data from remote areas to centrally based researchers, deliver feedback, and capture all interactions in a database. This Research Topic, “*Online data collection for human neuroscience: challenges and opportunities*”, highlights the progress that has been made toward improving the user friendliness, data quality, tool capabilities, and potential clinical applications of remote research.

Cognitive testing formerly required expensive, time-consuming, in-person administration by a trained professional. Since around 2000, there has been a large growth in online computerized cognitive testing. Recently, with the new focus on “disease modifying therapies” being suggested to benefit very mild Alzheimer's disease (van Dyck et al., 2023), there is a new focus on of the importance of early detection of brain disease affecting cognitive functions, with the hope that deterioration can be slowed or halted (Ashford et al., 2022).

Further, it is increasingly recognized that online testing can provide the needed precision, scalability, access, and reduced cost for both screening and assessment of cognition early in the course of cognitive deterioration. These requirements raise important issues in clinical practice, such as having resources to plan for disability or initiate health promoting behaviors to slow or reverse cognitive decline. Clinicians may not be confident that cognitive tests are sufficiently validated or reliable for use in individuals, and there are concerns about assuring responsible use and ensuring adequate understanding of potential problems when impairment is discovered, including discrimination and stigma (Rosen et al., 2022). This volume provides important perspectives of online testing and presents examples of online tests, which have the capacity to greatly improve and extend cognitive assessment, both for earlier detection of impairment and precisely determining severity, which can assist clinicians to diagnose such conditions sooner and more accurately.

In the article by Tomczak et al., “*What over 1,000,000 participants tell us about online research protocols*”, the authors note the growing adoption of tools for online research and describe a tool for creation and hosting of online studies, “Gorilla Experiment Builder” and a platform for holding data, “Prolific”, which contains data from “millions of participants and half a million studies”. Analysis of this data demonstrates critical insights into this type of study for informing future research development. They report frequency of use, sample sizes, study durations, and participant engagement metrics on this large dataset.

The article by Staggs and Mills-Finnerty, “*Protocol optimization and reducing dropout in online research*”, discusses the impact of optimizing protocols on the social engagement and retention of study participants. The discusses strategies for improving engagement and data quality and provides important tips for remote research.

The article by Kurzawski et al., “*EasyEyes—A new method for accurate fixation in online vision testing*”, provides critical discussion of central issues in remote assessment, where the participant is looking and attending, when there is no one monitoring the behavior of the participant. The EasyEyes open-source software is able to reliably and accurately achieve fixation, eliminating the need for in person gaze-tracking and testing large, diverse populations.

The article by Clifford et al., “*Episodic memory assessment: effects of sex and age on performance and response time during a continuous recognition task*” describes a simple implementation of a 2-min test, MemTrax, which has been taken over a million times and is widely used as a screen for memory dysfunction. The analyses show how a large quantity of data can be accumulated and analyzed to provide specific parameters that relate to the function of various brain mechanisms, similar to reports of neuropsychologists, including a novel insight into response time distribution changes with age.

The article by Woods, “*The California Cognitive Assessment Battery (CCAB)*”, describes a comprehensive cognitive battery for neuropsychological assessment with 15 non-verbal and 17 verbal subtests, for remote administration, using online connection with headphones and supervision by a remote examiner. Analysis of data from a population of patients, including individuals with a history of COVID infection, provides data for comparison of different environments for classifying mild-cognitive impairment, showing that the CCAB has the capability of providing high-quality assessments for a wide-variety of subjects in diverse settings.

The article by Zhao et al., “*Using MemTrax memory test to screen for post-stroke cognitive impairment after ischemic stroke: a cross-sectional study*”, demonstrates the great potential clinical utility of a simple online cognitive test for clinical assessment, follow-up, monitoring, and case management for individuals who have suffered cognitive impairment after a stroke. The study further shows the 2-min MemTrax test performs at least as well as the MoCA (Montreal Cognitive Assessment, which takes over 10-min) in subjects evaluated with the Clinical Dementia Rating scale.

The article by Binoy et al., “*Remote assessment of cognition in Parkinson's disease and Cerebellar Ataxia: the MoCA test in English and Hebrew*”, provides a comparison of remote administration of the MoCA and online scores with traditional in-person testing, showing that the scores from these two different testing modalities do not differ. Further, the online assessments in two patient groups, Parkinson and cerebellar ataxia patients, both showed impairment on the online assessments relative to controls, demonstrating the clinical utility of remote testing.

At this time, there is an increasing number of online cognitive assessment tools available, with over 40 tests either exempted or cleared by the FDA for non-diagnostic use, and there are many others yet to be so evaluated. The articles presented in this volume show that online cognitive testing has developed to the point where tools are ready for widespread use to increase the availability of neurocognitive screening, assessment, and measurement to improve clinical accessibility.

## Author contributions

CM-F: Writing – review & editing. PB: Writing – review & editing. JC: Writing – review & editing. JA: Writing – original draft.
